# Editorial Letter

**Published:** 1846-10

**Authors:** Daniel Drake

**Affiliations:** Cincinnati


### THE WESTERN JOURNAL.

NEW SERIES.—VOL. VI.—NO. IV.

LOUISVILLE, OCTOBER 1, 1846.

### EDITORIAL LETTER.

CINCINNATI, Sept. 15, 1846.

TO MY COLLEAGUES—*Gentlemen:*—I fear you will repent of having desired me to send you something for the forthcoming number of our Journal. Not that I am likely to write anything offensive to you or our readers, nor anything injurious to the health of their or your patients, nor anything which, by *warming* up the feelings of either, would add to the intolerable heat of the season; but simply because I have nothing of much importance to say, and shall not, therefore, be able to write anything which can interest our readers.

### COUP DE SOLEIL.

Every 'now and then' our newspapers announce a death from 'sun stroke,' but not being 'physician in ordinary' to the 'sovereign' people, I have not had the good fortune to see any of the victims;—in fact, have never yet seen a patient of that class; nor one fatally affected from drinking cold water on a hot day. Why is it that 'our brethren' who are called to such cases do not report them? All our journals abound in 'cases' of disease of daily occurrence, too many of which present but little that is new; while nothing is rarer than a reported case of sudden death from either of the causes to which I have referred. The close of a long and burning summer seems to be a proper time for presenting this subject to the profession, and I beg leave, therefore, to request such of our readers as have seen cases of the sort to report them, either for the Journal, or as contributions to a work on which I am engaged.

### TYPHOID FEVER.

I have lately assisted in the *post-mortem* examinution of two patients, who died after several weeks of illness, with symptoms answering very well to Louis' description of Typhoid Fever, and *not* answering to the symptoms which characterize the typhus fevers which I formerly saw prevailing here. Both the patients were men in the earlier periods of middle life, and both were 'new comers' here; reminding one of the class of patients in the practice of Louis. In one, the inflammation of the glands of Peyer extended along the lower five feet of the ilium, and for half that distance towards the cæcum they were in a state of ulceration. The lowest four inches of the small bowels presented ulceration of the intervening mucous membrane as well as of the elliptical patches, and the parietes of the tube were greatly thickened. In the other case, this thickening did not take place, and the consequence was the sudden death of the patient, after the fever had nearly ceased and he seemed to be convalescent. His physician, one of my friends, left him in the evening with a pulse at 72, but soon after reaching home was recalled. The patient had been suddenly seized with extreme pain just above the symphysis pubis. As yet his pulse was not much affected, but by morning it had become small, weak, and frequent, and his skin was covered with a clammy sweat. I saw him in the afternoon, when his abdomen was slightly tympanitic, his pulse thready, his countenance Hippocratic, and he was ejecting fluids from his stomach, by a kind of hiccup, or paroxysmal regurgitation, precisely similar to that which brings up the fatal black vomit of yellow fever. At two o'clock the next morning he expired. On opening the abdomen, a quantity of serum and lymph, colored with feculent matter, was found in the right iliac region and pelvis. The omentum was engorged and cemented to the intestines, which were covered with soft membraniform lymph. On further examination we found a circular perforation of the ilium, large enough to receive the quill with which I am writing, about two feet above the ilio-cæcal valve. It had occurred in the centre of an ulcerated elliptical plate, and not the least effort had been made by the *vis medicatrix* to thicken or sustain the peritoneum, at that point. It did not even present any hyperæmia. A number of the glands of Peyer were inflamed, and several of them ulcerated; but the lesions were not of such extent but that the patient might have recovered, had not the perforation led to a suddenly fatal peritonitis. It is worthy of record, that the attending and consulting physicians of this patient, both highly intelligent and respectable members of the profession, suspecting, from the general symptoms, an affection of the glands of the ilium, had made repeated examination of the ilio-cæcal region, and the patient always answered that there was neither pain nor tenderness. It is also deserving of record that in our post-mortem examination of the other patient, in whom so much ulceration existed, extending, as I may here add, into the cæcum and colon, we found, in the intestines, the usual quantity of healthy fæces. The two facts taken together show, that absence of pain and soreness under pressure and the presence of healthy alvine discharges, may coexist with fatal lesion of the glands of Peyer; and that the diagnosis of such cases, is, therefore, of a very doubtful and uncertain kind. How many other cases of the same sort with these have occurred in the 'Queen City' this summer, I cannot say; but, from what I have heard, I suppose there have been several. Indeed, just before the death of these patients, I was called into consultation, by one of our most promising young physicians over a gentleman, of nearly the same age with the other two, who had manifest symptoms of an acute *typhoid* fever, with decided, even peritoneal affection, of the right iliac region. The early loss of fifty ounces of blood, which was buffy, arrested the disease. This fever I suppose to be of the same kind with that which has prevailed, more or less, for several years past in the interior and oldest settled parts of Kentucky; and may be among our *increasing* diseases. If so, it is entitled to great attention;—just as an invading army should be more vigilantly watched than one which is retreating. If, with the 'progress of years,' typhoid fever should replace our autumnal remittents, we should not, I fancy, gain very much by the change. I wish it were possible, (in a successful and pointed manner,) to direct the attention of our readers upon this form of fever; and especially upon its connexions with autumnal fever, which seems frequently to modify, and be modified by it. Can any one tell whether those cases of remittent bilious fever, which, in their latter stages, exhibit typhoid symptoms, are accompanied by lesions of the ilium?

### THE SUMMER.

It should be recorded that the present summer has been one of unusual heat and rain. I say *present,* for although it is now the middle of the first month of autumn, the thermometer still rises to 85° and 88°, and even 92°. Thus the heat has not only been intense, but protracted. Howbeit, so frequent and copious have been the showers, that the 15th of September never before presented the forests which overshadow the hilly ramparts of the Ohio in deeper and healthier green. In fact, the frequent falls of rain have counteracted the fervent heat—fruits of all kinds have been abundant, and the withering effects of drought are nowhere to be seen. In the diseases of the city and surrounding country, there has not, as far as I know, been anything remarkable.

### QUACKERY.

The Queen City seems to have prostituted herself to the foul embraces of empiricism. Behold the gorgeous and glittering TEMPLE OF QUACKERY. On its dome there sports a gigantic black snake, fit emblem of cunning; and a silly coot (still fitter emblem of credulity,) is fluttering into the opening jaws of the wily fascinator. Let us enter the upper halls of the mansion of imposture.

Turn to the east; there is the den of the '*Reformed Medical College of Ohio,*' where doctors are manufactured out of the *raw* material. (N. B. The wool may be either coarse or fine, black, white or grey: the rolls warranted equally good and of the same size.) Now turn to the west; there is the den of the rival "*Eclectic Medical Institute,*" whose lathe can *turn* dunces into doctors, and not destroy the natural grain of the wood! The worthies who labor in these precious estalishments agree in one thing only, that of slandering the regular profession; beyond this, they show their impartiality by villifying, that is, telling the truth on, each other. I am meditating a plan by which to give a new impulse to their honorable emulation.

It is my good fortune to possess the only copy, in America, of a rare book of 1200 pages, published in London, in 1693, under the following comprehensive title:

"*Seplasium: the Complete English Physician; or, the Druggist's Shop opened. Explicating all the particulars of which medicines at this day are composed and made. Showing their various names and natures, their several virtues, preparations, uses and doses, as they are applicable to the whole art of physic, and containing above six hundred chemical processes. A work of exceeding use to all sorts of men, of what quality or profession soever; the like not hitherto extant. In ten books.* By WILLIAM SALMON, Professor of Physic, near Holburn Bridge, London. *Multa multuraque.*"

Now, the possession of this scarce and extraordinary work, by either the Reformed College, or the Eclectic Institute, would at once establish its supremacy over the other; and I hereby offer it as a premium to the one which can in the shortest time transform a hostler into a doctor; the prize to be adjudged by a committee of *gentlemen.*

Let us descend to another floor of the temple. Here, in an out-of-the-way corner, is the STEAMERY. But how deserted! Its fires are smouldering, and the distillery of 'No. 6' dribbles only *guttatim;* and yet the supply is equal to the demand. Compelling a sick girl to drink a quart of lobelia and then a gill of the tincture of red pepper, in a single night, while lying surrounded with ears of boiled corn, and winding up in the morning with a dose of 'No. 3,' is no longer the fashion: such methods of curing an inflammation of the brain or stomach are now condemned by the Court of Empiricism; and it has been discovered that a very different plan should be pursued. Let us enter the magazine where the new munitions are elaborated. But hold! We can't get in! It's a mere closet, with tiny boxes, and pills of the size of a millet seed at the end of a dry summer. A millionth of a grain of the extract of aconitum napellus in each box! A tenth of the ten millionth of a drop of the juice of atropa belladonna in that little vial. One pill in the morning, and one drop of the solution at night! No more steam polypharmacy! No more drenching, stuffing, and pistoning with herbs and roots. *That* was all very *vulgar—this* is very *refined:* suited to the character and constitutions of ladies and gentlemen—the learned and intellectual—above all, to the convenience of clergymen and pious ladies—whose pocket magazines will enable them to dispense to the poor, and to such of the rich as may be ashamed to visit the temple of quackery. Thus will the blessings of HOMŒOPATHY, like honey dew, fall equally on the leaves of the towering yellow poplar —*softest* of trees, and the humble elder—*greenest* of shrubs.

But let us pass on to another kennel. Its walls and ceiling and floor are wet and cold; but don't feel afraid to enter. You may have pleurisy, or rheumatism, or consumption; but "never mind"—sit down and receive the cold douche, or lie down and wrap yourself in the cold and dripping sheet. 'Steam' and hot 'chunks' *used* to be the *proper* remedies, but the college of quacks has made a new degree. In the days of Moliere, it could only transfer the liver from the right side to the left. Send round the book of HYDROPATHY. Let your friends be warned against all family physicians—all regular doctors, with their pedantic prescriptions and long bills! Be equally on your guard against homœopathy. Rely on water only and alone. Nothing can live *without* water—and nothing can die *with* it.

But we must move on to the next kennel, and a very genteel and quiet place it is. Look at the simplicity of its furniture—a chair and sofa. No steam baths—no cold water baths—no bundles of leaves and bark—no jugs of 'No. 6;' not even the miniature vials of homœopathic elixir, are allowed to profane the mysterious dormitory! Its very air is etherial—its light magnetic; and spiritualities gambol in the beams like gnats in the purple rays of the setting sun. All is intellectual and sublime. But let us come to the work. The necromancer takes his chair; the necromancee her sofa—their eyes meet each other,—he darts a magnetic glance—her eye-lids fall,—a quiver agitates her lips, and she softly reclines, in sleep of body but not of mind. 'A change has come o'er the spirit of her dream.' Her soul, disengaged from its frail tenement of clay, explores the caverns of the earth, and the lowest pools of the great deep; descends into hell, then rises to heaven; and, bringing tidings from both to him who thus set her free, is prepared, at his bidding, to enter the foul abodes of disease in the bodies of foul men, inspect the foul organs, with prying eyes, and report to her master the hidden cause of every foul infirmity. The patient thus inspected may now be placed under treatment. Put him on the sofa—let the doctor point his finger to the disordered organ—keep quiet till he gives the healing glance, and all will then be well. But we must not linger in this spiritual saloon, when so many *spirituous* kennels remain unexplored in the basement below. See how the *dogeries* are ranged around—lighted only by lamps—and labelled over every door 'No Admittance.' These are the secret vaults. What strong odors fill the air. Look at the inscriptions of the different doors:—"Challenge to the World!—*Dr. Cullen's Indian Vegetable Panacea.*" "The great Medicine of the day—*Dr. Swayne's Compound Syrup of Wild Cherry..*" "Coons! Coons! Coons!—*McKenzie's Corn Destroyer.*" "All opposition vain—*Bristol's Compound Syrup of Sarsaparilla.*" "Great cure for Consumption—*Dr. Duncan's Expectorant Remedy.*" "By the Queen's Patent--*Triumphant Success of Buchan's Hungarian Balsam of Life.*" "New Discovery —*Zanone's Hair Powder.*" "*Jayne's Life Preservative and Expectorant.*" "Fever and Ague, Chill Fever, Dumb Ague, Intermittent and Remittent Fevers, and all the various forms of Bilious Diseases speedily and thoroughly cured, *by Dr. Osgood's India Cholagogue!!*" "Salter's Ginseng Panacea—*The great remedy for Coughs, Colds, Influenza, Bronchitis, Asthma, Consumption, Pain in the Side and Breast, and all other Affections of the Lungs.* Approved by the Faculty of three Continents." Reference to twenty-seven gentlemen and ladies in Cincinnati, including "Dr. Drake!"

But what means the sign over that retired and central cell? A quill from the wing of a crow—cunningest and blackest of birds! Let us peep through the key-hole. Ah! there sits the scholar of all the subterranean kennels. See how nimbly his fingers move, and look at the sibylline leaves as they fall from his pen—infallible boluses—dulcified panaceas—tasteless catholicons—warranted specifics—renowned, restorative regenerators—reproducers of teeth in old age—universal resuscitators from every kind of apparent death! All attested and dignified by the names of eminent physicians and surgeons, now in their graves—Hunter, Cullen, Buchan, Wistar and Rush. And look at the blank certificates, ready to be filled up with spurious or forged names, by the manufacturers in the surrounding kennels! A discount to those who purchase by the ream; and no additional charge for filling up, to those inventors who do not know how to read and write.

Such, gentlemen, is a hasty sketch of the Temple of Quackery, which graces the Queen City. If its priests who minister at its altar are many, its votaries 'may be called legion.' They are no longer the uneducated and vulgar, but contrariwise, the cultivated, affluent and refined. In the midst of the attendance of well qualified and respectable physicians, both gentlemen and ladies have the nostrums of quacks smuggled into their apartments. Invalids have their carriages stopped half a square from the door of an empiric, and sneak to it on foot, lest some one (who is perhaps at the same time under the care of another quack) should chance to see them. Many, however, have grown quite shameless, and avow their preference of empiricism over science. Its harvest is undoubtedly very great; for those who follow it grow rich, while many deserving physicians 'live but from hand to mouth;' and others, too proud to grapple with knaves and impostors for the patronage of an *enlightened* community, are retiring to the country, and intend to give up physic for farming.

When I began this epistle I thought of some other matters on which I might say a little, but as you are undoubtedly tired, and "so am I," they will be kept back for another letter, till when,

I remain your obedient servant,

DANIEL DRAKE.

